# Recent developments in treatments for eating disorders

**DOI:** 10.1016/j.neurot.2025.e00773

**Published:** 2025-10-27

**Authors:** Alexandra R. Allam, Evelyn Attia

**Affiliations:** a Columbia University Irving Medical Center, 1051 Riverside Dr, New York, NY 10032, USA; b New York State Psychiatric Institute, 1051 Riverside Dr, New York, NY 10032, USA; c Weill Cornell Medicine, 1300 York Ave, New York, NY 10065, USA

**Keywords:** Eating disorders, Anorexia nervosa, Bulimia nervosa, Binge-eating disorder, ARFID, Neuromodulation

## Abstract

Eating disorders, including anorexia nervosa (AN), bulimia nervosa (BN), binge-eating disorder (BED), and avoidant/restrictive food intake disorder (ARFID), are serious psychiatric illnesses treated primarily with psychotherapy focusing on eating behaviors. Pharmacotherapy is recommended when psychotherapy is insufficient or unavailable, or when medication treatment is preferred by the patient. Differing psychotherapeutic approaches are used depending on the illness. Family-based treatment has demonstrated utility in adolescents with AN and BN. Eating disorder-focused cognitive behavioral therapy (CBT) is consistently helpful in individuals with BN and BED. Adaptations of CBT appear promising for the treatment of ARFID. Only two medications have received FDA approval for the treatment of eating disorders – fluoxetine for BN and lisdexamfetamine for BED. Existing treatments are not universally effective, and relapse rates are still elevated among those who do respond to treatment. Psychotherapies such as the habit-interrupting REACH ​+ ​for AN and biological treatments including neuromodulation techniques that target specific brain regions implicated in the development and maintenance of eating disorders warrant further study.

## Introduction

Eating disorders are a group of serious psychiatric illnesses characterized by disturbances in eating behaviors and associated with a myriad of medical and psychological impairments. The Diagnostic and Statistical Manual of Mental Disorders, 5th Edition (DSM-5) [1] lists six distinct Feeding and Eating Disorders: Anorexia Nervosa (AN), Bulimia Nervosa (BN), Avoidant/Restrictive Food Intake Disorder (ARFID), Binge-Eating Disorder (BED), Pica, and Rumination Disorder. In addition, several patterns of eating problems associated with impairment that do not meet full threshold criteria for the formally recognized diagnoses are described in a category of Other Specified Feeding and Eating Disorders.

Treatments for eating disorders include psychotherapy and medications that aim to improve eating behaviors and other core symptoms of specific eating disorders. The complexity of eating disorders often leads to treatments delivered by multi-disciplinary teams including medical providers, therapists and dietitians. Individuals with eating disorders may receive treatment in settings that vary in intensity (i.e., degree of available supervision and clinical monitoring) from outpatient to residential or inpatient, depending on the severity of medical or psychiatric symptoms during illness episode.

The purpose of this review is to describe evidence-based treatments for eating disorders with emphasis on recent developments in treatment research. Compared with other recent reviews that have examined recommended interventions for eating disorders [2], the current study aims to comprehensively review both medication and psychotherapy interventions, as well as treatments that are under development and show promise in their efficacy. PubMed and PsycINFO databases were systematically searched for English-language studies of treatment for eating disorders by using a combination of keywords such as “Anorexia Nervosa,” “Bulimia Nervosa,” “Binge-Eating Disorder,” “Avoidant-Restrictive Food Intake Disorder,” “Randomized Controlled Trials,” “Cognitive Behavioral Therapy”, “Family Based Treatment”, “Interpersonal Psychotherapy”, “CBT-E″, “Pharmacotherapy”, “Evidence-Based Treatments,” “Neuromodulation,” “TMS”, “DBS”, and “tDCS” in conjunction with appropriate Boolean operators (AND, OR) between 2018 and 2025. In addition, the references of the included studies were thoroughly examined to encompass all pertinent research. Randomized clinical trials (RCTs) and Systematic Reviews were prioritized for inclusion.

## Anorexia nervosa

Although descriptions of individuals who refrained from eating as part of religious or spiritual beliefs and developed medical and behavioral features that resemble current day AN date back to the Middle Ages, self-imposed food restriction linked to psychological symptoms was first described and termed “anorexia nervosa,” by William Gull in 1873 [3]. From the late 1800's through 1952, when the first edition of the *Diagnostic and Statistical Manual* (*DSM-I*) [4] was released and AN was included as the first and only eating disorder diagnosis at the time, professionals in the medical and psychological fields began characterizing AN's physical and psychological symptoms [5,6]. While theories about the underlying mechanisms of AN have evolved, the early descriptions of the disorder mirror the phenomenology described in the most recent, fifth edition of the *DSM* (*DSM-*5-TR*)* [7,8].

AN is now recognized as a brain-based disorder characterized by marked and persistent restriction of energy intake relative to requirements leading to significantly low body weight, intense fear of gaining weight or becoming fat, or persistent behavior that interferes with weight gain, and disturbance in the way in which one's body weight or shape is experienced [7]. There are two subtypes of AN with which individuals with the disorder can be diagnosed: restricting type (AN-R) and binge-eating/purging type (AN-BP).

Restricted energy intake, low body weight, and compensatory behaviors to prevent weight gain present in individuals with AN result in commonly observed medical complications [[7], [8], [9]]. Medical features associated with AN include cachexia, sometimes accompanied by lanugo; vital sign changes consistent with metabolic slowing including bradycardia, hypotension and hypothermia; blood cell deficiencies such as anemia and leukopenia; electrolyte imbalances; cardiac changes including arrhythmias, mitral valve prolapse and pericardial effusion; structural brain changes including decreases in gray matter and increases in ventricular size; endocrine effects including decreased levels of reproductive hormones, absence of menstruation (amenorrhea) or delay of menarche in prepubertal females; low bone density that can result in osteopenia or osteoporosis; gastrointestinal effects including slowed gastric emptying, abdominal pain, and constipation; and liver transaminitis and pancreatitis [7]. Suicide risk is also elevated in AN with rates of 12 per 100,000 per year [10]. The potentially life-threatening medical complications together with suicide risk contribute to the all-cause mortality rate for individuals with AN being significantly elevated. In fact, mortality risk for AN is the highest of any psychiatric illness with a crude mortality rate at approximately 5 ​% per decade of illness [7, 10].

Despite the long-standing recognition of AN as a serious disorder and the frequent severity of its presentation, treatment progress has been slow with most RCT's finding little-to-no effect of medication vs placebo. An exception has been the atypical antipsychotic medication, olanzapine, that is associated with modest weight gain compared with placebo in adult patients with AN. Behavioral treatments for AN are useful for acute weight restoration, but leave many individuals, especially adults, vulnerable to relapse following acute treatment. More recent mechanism-based treatments are under development, including behavioral treatments that target habit-learning and neuromodulatory approaches that target brain circuits hypothesized to be engaged during food choice tasks in AN.

### Psychopharmacology

Despite efforts to identify effective pharmacotherapy for AN, there are currently no guideline-recommended medications. Initially, medication trials focused on the symptom-overlap between AN and depression (e.g., low energy, sleep disturbance, suicidality) and anxiety disorders (e.g., intrusive preoccupations, fears including of weight gain, generalized anxiety), hypothesizing that anti-depressant medications might be helpful to individuals with AN. Many small RCT's examined antidepressant medications vs placebo and found no benefit associated with medication promoting weight gain or improving psychological symptoms in individuals with AN [11]. However, olanzapine, an atypical antipsychotic medication, has shown promise in promoting modest weight gain [[11], [12], [13]].

#### Olanzapine

Several studies have evaluated olanzapine in the treatment of AN [12,[14], [15], [16], [17], [18], [19]]. In the largest RCT to date, 152 outpatient individuals with AN achieved a mean increase in body mass index (BMI) of 0.259 (0.051) per month vs 0.095 (0.053) with placebo (*p* ​= ​0.03), the equivalent of 0.7 ​kg/month vs 0.26 ​kg/month for an average-height patient (e.g., 165 ​cm), without adverse effects such as hyperglycemia and hyperlipidemia [12]. Less certain is the impact of olanzapine on psychological symptoms of AN. Attia et al. [12] found improvements in somatic symptoms associated with olanzapine but not improvements in obsessionality or other psychological symptoms. An RCT by Bissada et al. [15] that included 34 women with AN participating in day treatment found a significant difference in rate of improvement of obsessional symptoms associated with olanzapine vs placebo, and a smaller pilot study by Mondraty et al. [19] similarly identified a decrease in ruminative cognitions associated with olanzapine.

### Biologically-based treatments under development

There is increased recognition that AN is a biologically based disorder; recent technological advances have led to new understanding of possible illness mechanisms for AN and associated treatment developments have targeted these hypothesized mechanisms. Behavioral studies using a computerized Food Choice Task have shown that individuals with AN are significantly less likely than healthy volunteers to select high-fat foods [20] and that the performance on the computer task significantly correlates with actual food intake [21,22]. Neuroimaging research using the same task has found that when making food choices, individuals with AN engage dorsal striatal brain regions, as compared with healthy volunteers [21,22]. Additionally, individuals with AN exhibit differential functional connectivity between a specific region of the right dorsolateral prefrontal cortex (DLPFC) and dorsal striatum when making decisions about high-fat foods than when making decisions about low-fat foods [22], suggesting that frontostriatal circuitry may underlie restrictive food choice in AN. Repetitive transcranial magnetic stimulation (rTMS) has been used as a means to target dorsal striatal circuitry both to test this hypothesis and to treat the underlying behavioral disturbance.

#### Neuromodulation

Multiple case reports and case series describe rTMS to be associated with clinical improvements in AN [[23], [24], [25], [26], [27]]. A small trial that compared high-frequency repetitive transcranial magnetic stimulation (HF-rTMS) to sham using crossover design found that active treatment to a region of the right DLPFC was associated with change to food selection using the Food Choice Task among adult inpatients with AN; specifically, that patients were more likely to select higher fat foods following the single session treatment [28]. Another sham-controlled RCT of rTMS in AN found that, among 17 participants who received 20 sessions of real HF-rTMS to the left DLPFC experienced greater improvements in BMI (after adjusting for baseline BMI: mean BMI increase of 0.11 post-treatment and 0.28 ​at follow-up in the rTMS group compared with decrease of 0.08 ​at post-treatment and increase of 0.04 ​at follow-up in the sham group) and eating disorder symptoms [29]. Between-group effect sizes were small at end of treatment (EOT) and short-term follow-up but became more pronounced over longer-term follow-up, whereby change in BMI (after adjusting for baseline BMI) at 18-months post-randomization was an increase of 2.13 in the rTMS group compared to 0.76 in the sham group, suggesting that additional improvement may occur following treatment [30].

Other methods of neuromodulation, including transcranial direct current stimulation (tDCS) and deep brain stimulation (DBS), have also been considered as possible therapeutic approaches for treatment-refractory AN with most delivering a fixed, weak current and generating subthreshold changes to targeted neurons. tDCS can be used to increase or decrease neuronal activity and, in an open-label pilot study, 5 of 7 individuals with AN who received 10 sessions of excitatory tDCS over the left DLPFC reported short-term decreases in measures of eating disorder and depression symptoms [31]. In an RCT involving 23 adolescents with AN, tDCS offered together with treatment as usual was compared to a family-based therapy, with a greater increase in BMI over time and at 1-month follow up in the group receiving tDCS (mean percentage of BMI improvement ​= ​13.3 ​% [±9.4]) compared with family-based therapy (mean percentage of BMI improvement ​= ​4.2 ​% [±5.7]) [32].

More invasive than tDCS, DBS, a procedure in which electrodes are surgically implanted and deliver electrical pulses to target brain regions, has also been considered for study in AN. Case reports [[33], [34], [35]] and a small case series [36] of individuals with AN describe improvements in mood, weight, and eating disorder symptoms associated with DBS [[33], [34], [35]]. Subsequent open-label trials of DBS implantation in the subcallosal cingulate and nucleus accumbens reported improvements in some eating disorder symptoms and weight across follow-up [37,38]. Specifically, one study found BMI increased by 3.51 from baseline to 12-month follow-up [37], and another showed mean BMI increases of 2.28 post-surgery at 6-month follow-up and 4.72 ​at 2-year follow-up, with patients with AN-R having more significant improvements in weight than those with AN-BP [38]. Although case reports and clinical studies using DBS suggest general tolerability and an adequate safety profile for the procedure, the use of DBS for AN remains controversial and its acceptability and efficacy are in need of further study with control conditions [39].

#### Psilocybin

Features associated with AN including cognitive rigidity, high anxiety, and obsessionality, as well as documented disturbances in serotonin (5-HT) function in AN have led to an interest in whether psychedelic compounds such as psilocybin may be helpful in symptom improvement for individuals with AN. While the literature examined for this review found no published RCT's examining psilocybin in AN, a single phase 1 (i.e., safety) open-label feasibility study reported on 10 adult females with AN or partial remission AN who received a single 25-mg dose of synthetic psilocybin together with psychological support [40]. Psilocybin was acceptable for most participants and weight concern, as measured by the Eating Disorders Examination (EDE), decreased significantly with *t*-tests demonstrating change from baseline (day −1) to 1-month (P ​= ​0.036, Cohenʼs d ​= ​0.78) and 3-month (P ​= ​0.04, d ​= ​0.78) follow-up, with a medium-to-large effect [40]. This study did not find significant effect of psilocybin on BMI over time, with great variability between participants on BMI trajectory. This study was also limited by lack of a control condition.

#### Dronabinol

Given that the cannabinoid system is involved in modulating appetite and metabolic processes, the orexigenic and anabolic effects of dronabinol (a synthetic cannabinoid) have been studied in AN. In one randomized, double blind, controlled crossover study [41] examining the effects of dronabinol on weight gain and psychological eating disorder symptoms in 25 females with severe and enduring AN, results showed that dronabinol was well tolerated and significantly predicted weight gain. Dronabinol was associated with 0.17 ​kg greater weekly weight gain compared with placebo and 0.66 ​kg total greater weight gain over 4 weeks than weight gain during placebo [41]. However, dronabinol was not significantly related to attitudinal and behavioral traits related to eating disorders and, though plasma leptin levels rose to 15 ​% above placebo during the dronabinol phase, these levels returned to baseline after the last dose of dronabinol [41]. However, there is limited other research examining dronabinol in AN, particularly with respect to its effects on psychopathology [42].

### Psychotherapy

Several behaviorally focused psychotherapies are effective at improving weight and associated psychological symptoms in AN. Family-based treatment (FBT) has a strong evidence base for adolescents with AN, and several psychotherapeutic approaches are associated with modest clinical benefit in adults with AN with no one treatment type being superior to the others [12,43]. Recently, mechanism-based treatment research has contributed to the development of novel treatments for AN that aim to interrupt habit-like behaviors relevant to differences in brain region engagement that has been identified in AN [44,45].

#### Family-based treatment (FBT)

FBT first emerged as a treatment for AN more than 30 years ago at the Maudsley Hospital in London [46] but was manualized and studied more comprehensively by James Lock and colleagues in the early 2000's [47,48]. Aiming to empower families to help refeed their children with AN, the treatment includes psychoeducation, weight restoration with monitoring, and a transition to returning autonomy back to the adolescent before treatment completion. In a study of 121 adolescents with AN comparing FBT to individually delivered adolescent-focused therapy (AFT), BMI percentile achieved was higher in FBT group at EOT, as was rate of partial remission and eating disorder symptom improvement as measured by the EDE. Full remission rate was significantly higher in the group receiving FBT at both 6- and 12-month follow-up assessments [48].

A number of RCTs have extended the early work on FBT, with studies comparing FBT to parent-focused treatment (PFT) [49,50], where a therapist meets only with the parents while a nurse monitors the adolescent; separated family therapy (SFT) [51,52], where the adolescent and parents are each seen separately by the therapist for the treatment; and multi-family therapy (MFT) [53], which focuses on families drawing support from other families in group settings. Results from these RCTs have demonstrated that FBT and PFT both successfully lead to increases in weight, positive affect, and decreases in dietary restraint and negative affect in patients with AN over the course of treatment [50], and that FBT is superior to SFT in short-term outcomes, but that there are no significant differences in treatment outcomes between FBT and SFT at 5-year follow-up [52]. Results from the RCT comparing FBT to MFT showed significantly better outcomes at 18-month follow-up in the MFT condition than the FBT condition, with 57 ​% reaching intermediate or good outcomes in the FBT condition and 78 ​% in the MFT condition [53].

Still, only 33–42 ​% of adolescents who receive FBT remain fully recovered 3–4 years following treatment [54] and many with AN do not have access to FBT providers or do not have the family support necessary to utilize this approach. Therefore, more recent RCTs have investigated ways to improve outcomes and increase accessibility. Although augmenting FBT with intensive parental coaching for early non-responders did not improve remission rates [54], the feasibility of an online guided self-help program version of FBT and FBT delivered via videoconferencing have been demonstrated with more research needed to fully evaluate the efficacy of these methods of delivery [55]. Moreover, a recent meta-analysis examining variations of FBT offered either conjointly to parents and children with AN, to just parents, or separately to parents and to the child, found that FBT was most effective when offered either to parents alone, or separately to parents and to child than conjointly in terms of weight outcomes (*g* ​= ​−0.42, 95 ​% CI [−0.73, −0.11]) and rates of remission at the end of treatment (RR ​= ​0.56, 95 ​% CI [0.38, 0.83]) [56].

#### Enhanced cognitive behavioral therapy

While FBT is helpful for the treatment of AN in adolescents, there is less certainty about effective strategies for treating adults with AN [57]. However, enhanced cognitive behavioral therapy (CBT-E), a version of CBT designed for people with eating disorder psychopathology and founded on the transdiagnostic cognitive behavioral model of eating disorders, has attempted to fill this gap [58,59]. A multi-center RCT conducted in Germany examined the efficacy and safety of treating adults with AN using CBT-E, compared to an alternative, manualized treatment for AN called “focal psychodynamic therapy,” and to optimized treatment as usual in 242 adult females with BMIs of 15–18.5 [60]. Zipfel and colleagues [60] found that, for participants with a BMI below 17.5 ​at baseline, participants assigned to the CBT-E condition reached significantly higher mean BMI at EOT compared to those receiving focal psychodynamic therapy group and reported lower symptoms on the Structured Expert Interview for Anorexic and Bulimic Syndromes (SIAB-EX) [61] compared to those in both other conditions. In another study comparing CBT-E to the Maudsley Anorexia Nervosa Treatment for Adults (MANTRA) and Specialist Supportive Clinical Management (SSCM; an alternative to treatment as usual that combines clinical management and supportive psychotherapy within sessions) [62], no statistically significant differences between treatments were found with regard to BMI change, or changes in psychopathology and impairment with mean increases in BMI, and significant decreases in depression anxiety, and stress, clinical impairment, and eating disorder symptoms being associated with all three study conditions. Notably, however, 59 ​% of CBT-E participants achieved a physically healthy weight (BMI >18.5) at 12-month follow-up, compared to 47.5 ​% in SSCM and 44 ​% in MANTRA [62].

### Psychotherapeutic treatments under development

As neurobiological research has focused on the persistent maladaptive food choice behaviors characteristic of AN, and as disturbances in brain circuitry associated with these behaviors have begun to be identified in individuals with AN, it has become possible to target hypothesized mechanisms in the development of novel psychotherapeutic treatments. Specifically, a model has been proposed that restrictive eating behaviors seen in AN are learned sequences of behaviors that become automatic responses to specific cues (i.e., habits) [44,63] supported by results of a food choice computer task that has found across many samples that individuals with AN have predictable food choice patterns, consistently avoiding high-fat foods [21,22] and that performance on the Food Choice Task closely correlates with actual intake [20]. Additionally, the same task when performed along with brain imaging demonstrates engagement of dorsal striatal brain regions in individuals with AN and not in healthy control samples [22]. Regulating Emotions and Changing Habits (REaCH) is a treatment based on habit reversal therapy [45,64] that aims to assist in cue identification together with interruption of behavioral routines experienced as automatic by those with AN [44]. In a proof-of-concept study, ReaCH was compared with Supportive Psychotherapy in 22 hospitalized patients with AN one week following hospital admission and was associated with significantly lower Self-Report Habit Index scores, as well as lower eating disorder symptoms as measured by the EDE-Q Global score at EOT [44]. Since its inception, ReaCH has been modified and optimized as Relapse Prevention and Changing Habits (REACH+) to also focus on relapse prevention post-acute treatment, though further research is needed to establish its efficacy [45].

## Bulimia Nervosa

BN is characterized by recurrent episodes of binge eating followed by inappropriate compensatory behaviors to prevent weight gain, including purging via self-induced vomiting, misuse of laxatives, diuretics, or diet pills, fasting, or excessive exercise which occur on average at least once per week for a minimum of three months [7]. Individuals with BN also experience self-evaluation that is unduly influenced by body shape and weight, but maintain normal or above-normal BMI.

### Psychopharmacology

In clinical trials for BN, most antidepressant medications that have been tried are more effective than placebo in reducing the frequency of binge-eating and purging episodes [13]. A recent meta-analysis evaluated results from RCTs testing antidepressants in the treatment of BN and found in addition to fluoxetine, selective serotonin reuptake inhibitors (SSRIs) of fluvoxamine and amineptine have been shown to reduce some BN symptoms, as did tricyclic antidepressants imipramine and desipramine and the multimodal monoamine oxidase-A inhibitor, brofaromine [11]. However, to date, only fluoxetine is approved by the United States Food and Drug Administration (FDA) for use in BN.

#### Fluoxetine

Fluoxetine has been tested in 10 RCTs and has been found to outperform placebo in reducing binge-eating and purging episode frequency in nine of these trials [11]. A large study found a dosage of 60 ​mg was more effective than placebo while 20 ​mg was not in decreasing the frequency of weekly binge-eating and vomiting episodes, as well as improving depression, carbohydrate craving, and pathologic eating attitudes and behaviors in individuals with BN [65], leading to current consensus in practice guideline recommendations that 60 ​mg fluoxetine be considered for BN either initially or if there is minimal response to psychotherapy alone after six weeks of treatment [66]. Fluoxetine has also been found to be effective when administered in conjunction with CBT [67]. For patients who have previously not responded to CBT or interpersonal psychotherapy, fluoxetine alone has also been shown to be effective at reducing the frequency of binge eating and purging and in improving scores on the Three-Factor Eating Questionnaire and global EDE scores [68].

### Biologically-based treatments under development

#### Neuromodulation

A recent review of research investigating neuromodulation techniques (DBS, tDCS, and TMS) applied to the treatment of eating disorders found one study on tDCS in BN and several investigating TMS in BN or in BN and eating disorder not otherwise specified (EDNOS; categorized in DSM-5 as other specified feeding or eating disorder, OSFED) [69]. The one study included in this review that used tDCS in 39 adults (2 male) with BN indicated that tDCS is associated with a reduction in urges to binge-eat and increases in self-regulatory control following stimulation [69,70]. More research has been conducted using TMS and rTMS in BN and in combined samples of individuals with BN and EDNOS. In two studies examining rTMS in BN patients only, results indicated that rTMS to the left DLPFC may be associated with a reduction in binge-eating episodes after 15 sessions compared to placebo/sham [71], but not after 10 sessions [72] and does not have an effect on purging [69]. Other studies conducted on samples with heterogeneous eating disorders have similarly found rTMS to be associated with reduction in urges to binge eat and food cravings, but not on other symptoms characteristic of BN [69]. More research is needed examining BN-only samples to better understand the effects of neuromodulation techniques in the treatment of BN.

#### Topiramate

Although recent research has been more limited in evaluating topiramate in BN, two RCTs were conducted examining topiramate in this population that showed some clinical benefit to binge-eating frequency, although concern about weight loss associated with this medication has limited its utility. In one study, 30 outpatients with BN were treated with a starting dose of 25 ​mg of topiramate that was titrated to 250 ​mg/day (by week 6) in a 10-week randomized, double-blind, placebo-controlled study that took place in Germany [73]. This study showed topiramate was tolerated well. Moreover, compared with the placebo group, those who received topiramate showed significant reduction in binge eating and purging over the 10 weeks, but also had a significantly greater amount of weight loss from baseline to end of treatment (4 ​kg compared to 0.3 ​kg in placebo group; *p* ​< ​0.001) [73]. In the other randomized, double-blind, placebo-controlled trial examining topiramate in BN, 35 outpatients with BN receiving topiramate were compared to 34 outpatients with BN in the placebo group over 10 weeks [74]. In the topiramate group, participants were started at 25 ​mg/day and titrated by 25–50 ​mg/week to a maximum of 400 ​mg/day. Topiramate was associated with significant improvements in binge eating and purging in this study compared with placebo and was generally well tolerated [74].

### Psychotherapy

The American Psychiatric Association (APA) [75] recommends eating disorder-focused CBT for the treatment of adults with BN either alone or in conjunction with an SSRI. In addition to research supporting the efficacy of CBT for BN, studies have also found interpersonal psychotherapy (IPT) to be effective in the treatment of BN, with CBT for BN demonstrating faster results but continued improvement in IPT contributing to similar efficacy at 8-month follow-up [[76], [77], [78]]. A recent RCT has also found evidence supporting FBT in the treatment of BN in adolescents [79], which is consistent with APA [75] practice guideline suggestions that eating-disorder focused family-based treatment be used for the treatment of BN in adolescents and emerging adults.

#### CBT

Eating disorder-focused CBT is currently the gold standard and first-line treatment for BN [13, 75, 80]. Studies have found CBT to be more effective than waitlist control [81,82], psychodynamically-oriented supportive psychotherapy [83], and nutritional therapy alone [84] at reducing frequency of binge-eating and purging episodes in individuals with BN. RCTs comparing CBT with IPT have shown that CBT is more effective at reducing frequency of binge-eating and purging episodes in BN at EOT but not at follow-up [[76], [77], [78]]. Although the majority of research on cognitive-behavioral treatments for BN has examined CBT more broadly, RCTs examining the efficacy of the more recently introduced CBT-E in the treatment of BN have also been conducted. Fairburn et al. [85] found that two versions of CBT-E (a focused form targeting eating disorder psychopathology exclusively: CBT-Ef; and a more complex broad form targeting eating disorder psychopathology and additional problems that are theorized to maintain the disorder: CBT-Eb) when compared with waitlist control were associated with greater reductions in symptom severity among patients with BN and these reductions were maintained at 60-week follow-up assessment, at which point 61.4 ​% of patients with BN had a global EDE score less than one standard deviation above the community mean for adult females (i.e., below 1.74). A more recent proof-of-concept RCT investigating the acceptability, feasibility, and preliminary evidence of Metacognitive Interpersonal Therapy (MIT-ED) compared with CBT-E in a sample of participants with non-underweight eating disorders, including BN, found CBT-E was associated with a reduction in eating disorder symptoms, impairment, and binge eating [86].

#### IPT

IPT is a psychotherapy aimed at addressing four interpersonal problem domains: role disputes, role transitions, interpersonal deficits, and unresolved grief. IPT has been found to be less effective than CBT in its treatment of BN over the same duration, but to achieve similar results at follow-up [[76], [77], [78],^87^]. IPT is speculated to indirectly treat eating disorders by targeting the underlying interpersonal issues that maintain them and, therefore, by preventing further problems that might trigger the individual to rely on symptoms to cope [88]. An eating disorder-specific model of IPT (IPT-ED) has been developed to potentially improve the efficacy and speed with which IPT treats BN, but further research is needed to evaluate how this form compares to CBT [89].

#### FBT

One RCT examining FBT for BN [90] to individually administered CBT adapted for adolescents found FBT to be more effective at reducing binge-eating and purging episodes in 130 adolescents with BN at EOT and 6-month follow-up [79]. However, there were no statistically significant differences between CBT and FBT in this sample at 12-month follow-up.

## Binge-eating disorder

BED is characterized by recurrent episodes of binge eating, which are associated with eating much more rapidly than normal, until uncomfortably full, in the absence of physical hunger, and/or alone due to embarrassment about quantity of intake [7]. Individuals with BED also may feel disgusted, depressed or very guilty after a binge-eating episode and experience significant distress regarding binge eating [7]. Episodes of binge eating in BED must occur on average at least once per week for a minimum of three months and are not associated with recurrent use of inappropriate compensatory behaviors [7].

### Psychopharmacology

Medication trials in the treatment of BED have included antidepressants and medications that are effective at appetite suppression [13]. Across trials, most medications studied have been more effective than placebo at reducing the frequency of binge-eating episodes, although antidepressants have little-to-no effect on weight in individuals with BED [13,91]. Moreover, across studies, large placebo response rates have been found in the treatment of BED suggesting that non-specific factors may be helpful in treating BED [92].

#### Lisdexamfetamine

Among medications applied in the treatment of BED, lisdexamfetamine, a psychostimulant medication traditionally used to treat attention-deficit/hyperactivity disorder, is the only medication approved by the United States FDA for the treatment of BED [93]. Across three RCTs evaluating lisdexamfetamine in individuals with BED, lisdexamfetamine 50 ​mg and 70 ​mg demonstrated greater efficacy than 30 ​mg or placebo at reducing frequency of binge eating [11]. Lisdexamfetamine was also associated with a significant decrease in weight and BMI [94], and the optimal doses for improving general and specific eating disorder psychopathology and four-week cessation of binge-eating were 50 and 70 ​mg/day [11,95,96]. However, adverse events associated with lisdexamfetamine, including dry mouth, insomnia, and headache are common [11].

#### Topiramate

In addition to antidepressants and medications associated with appetite suppression, topiramate, an antiepileptic agent associated with weight loss, has been examined in two RCT's for individuals with BED and BMI ≥ 30 [97,98]. Compared with placebo, topiramate was associated with a significantly greater reduction in binge eating frequency, BMI, and weight compared with placebo. Medication side effects including slowed thinking, difficulty concentrating, and tingling sensation in the extremities limit the utility of topiramate for BED.

#### SSRIs

Several SSRIs have been tested for their utility in the treatment of BED. Compared to placebo, fluoxetine has been associated with a significant reduction in binge eating frequency, BMI, weight, and severity of illness among individuals with BED [99]. However, other studies comparing fluoxetine and CBT alone and in combination, as well as to placebo, found fluoxetine alone not to be superior to placebo in the treatment of BED, although fluoxetine in combination with CBT was more effective than fluoxetine alone or placebo [11,100,101]. However, in one study that compared fluoxetine to sertraline, both SSRIs were associated with a significant reduction in body weight, frequency of binge-eating episodes, and improvement in binge-eating behavior [102]. Sertraline has also been found to be effective in reducing the frequency of binge-eating episodes and BMI in other studies [11,102,103], though Brambilla et al. [104] found that among participants with BED, combination therapy with sertraline in conjunction with topiramate, CBT, and a diet of 1700 ​kcal (21 ​% proteins, 27 ​% lipids, 52 ​% carbohydrate) had superior effects compared to sertraline without topiramate plus CBT and a diet of 1700 ​kcal without topiramate. The APA [75] suggests that adults with BED who prefer medication or have not responded to psychotherapy alone be treated with either lisdexamfetamine or an antidepressant medication.

### Biologically-based treatments under development

#### Neuromodulation

There is a lack of research on specific neuromodulation techniques to treat BED perhaps given its more recent incorporation into the DSM-5 [1] and the relative efficacy of other main-line interventions. Although one recent study used TMS measures to assess the cortical excitability among patients with BED, there is limited research on rTMS as an intervention to treat eating disorder psychopathology in this population. However, research on patients with EDNOS and with BN have found that rTMS has been associated with decreased binge-eating episodes and food cravings [69], indicating rTMS may be useful in addressing core features of BED.

### Psychotherapy

As in the treatment of BN, both CBT and IPT have demonstrated efficacy in the treatment of BED [105].

#### CBT

In a recent meta-analysis of 79 trials of CBT in the treatment of BED, therapist-led CBT significantly outperformed waitlist control in remission rates and reductions of binge-eating episodes and was superior to active comparison treatments (any psychotherapy) on frequency of binge-eating episodes and cognitive symptoms in individuals with BED [106]. Moreover, therapist-led CBT was significantly more efficacious than medication on improving cognitive symptoms and decreasing binge-eating frequency, particularly at long-term follow-up [106]. Therapist-led CBT was more effective than IPT at reducing behavioral and cognitive symptoms of BED at posttreatment [106]. CBT for BED was also more effective than behavioral weight loss interventions in reducing behavioral symptoms of BED in individuals with comorbid overweight obesity [106]. Although self-help CBT for BED performed better than waitlist controls at reducing symptoms and had better remission rates at long-term follow-up, it was not more effective than active comparisons, and follow-up analyses between self-help CBT and active comparisons could not be performed [106].

#### IPT

IPT has been evaluated as an alternative psychotherapeutic intervention to CBT for the treatment of BED, with evidence supporting its efficacy in reducing binge eating compared to waitlist control [107]. Both self-help CBT and IPT have been found to be associated with greater remission from binge eating compared to behavioral weight loss interventions at 2-year follow-up, and IPT was associated with greater symptom reduction for individuals with low self-esteem and elevated eating disorder psychopathology [107].

## Avoidant/Restrictive food intake disorder

ARFID was first defined in the DSM-5 [1] and is characterized by restrictive eating in the absence of concerns about body weight or shape. Individuals with ARFID limit the types and/or amounts of food they consume due to lack of interest, aversive responses to taste, texture, smell or appearance of certain foods, or fear of negative outcomes associated with eating (e.g., choking), which results in persistent failure to meet appropriate nutritional and energy needs and/or psychosocial impairment [7].

### Psychopharmacology

To date, there have been no RCT's examining medication for ARFID and no medication has been approved by the United States FDA for its treatment [108]. A few case series and case reports have been published examining the use of olanzapine, mirtazapine, and buspirone in conjunction with psychotherapy to treat children and adolescents with ARFID, but their generalizability is limited due to the lack of systematic approaches to this area of research [109].

### Psychotherapy

Research is limited about treatments for ARFID, likely due to its having been only recently introduced into the DSM-5 and ICD-11. A recent scoping review by Willmott et al. [110] identified 50 publications describing treatments for ARFID since its introduction, nearly half of which (*n* ​= ​23) were single case studies and most of which (*n* ​= ​42) were studies of children and adolescents. Common treatment components included food exposure, psychoeducation, anxiety management and family involvement. Studies were limited by small and heterogeneous study samples, lack of validated measures used to describe outcomes with many studies assessing only physical health metrics (e.g. weight). A Cognitive-Behavioral Therapy for ARFID (CBT-AR) has been developed and published as a treatment manual, already helping the field build an evidence base regarding its use. Preliminary studies support that CBT-AR is feasible, acceptable, and appears to help both youth and adults with ARFID expand food choice [111,112].

## Discussion

Eating disorders, including AN, BN, BED, and ARFID are serious psychiatric illnesses; treatments generally include psychotherapy focused on eating behaviors. Medications are used in addition to psychotherapy when needed or preferred by the patient. Multi-disciplinary teams of providers including primary care, psychiatry, psychology, and nutrition are frequently assembled to coordinate comprehensive care for the medical as well as psychological features associated with these disorders. The evidence base supports various specific psychotherapeutic approaches for different disorders and populations. FBT that engages parents in recovery-focused treatment for youth with AN and BN has demonstrated utility in restoring weight in individuals with AN and in interrupting binge eating and purging behaviors in those with BN. CBT, with emphasis on challenging automatic thoughts that drive eating behaviors characteristic of illness, is consistently helpful in individuals with BN and BED. Adaptations of CBT that emphasize introducing novel foods appear promising for the treatment of ARFID. While these treatments help significant numbers of individuals with eating disorders, they do not help all, and improvements are sometimes short-term, leaving individuals vulnerable to relapse. Newer approaches, both psychotherapies and biological treatments, aim more specifically to target neurobiological disturbances and offer hope for interrupting illness mechanism. Psychotherapies such as the habit-interrupting REACH ​+ ​for AN and biological treatments including neuromodulation techniques that target specific brain regions implicated in the development and maintenance of eating disorders warrant further study.

## Author contributions

Alexandra Allam drafted the manuscript. Evelyn Attia assisted with drafting the manuscript and editing. All authors reviewed the manuscript.

## Declaration of competing interest

The authors declare the following financial interests/personal relationships which may be considered as potential competing interests: Alexandra R Allam reports financial support was provided by National Institute of Mental Health T32 Fellowship (MH096679). Evelyn Attia reports a relationship with Wolters Kluwer that includes: consulting or advisory. Evelyn Attia reports a relationship with Oxford University Press that includes: consulting or advisory. Evelyn Attia reports a relationship with Equip Health Inc that includes: equity or stocks. If there are other authors, they declare that they have no known competing financial interests or personal relationships that could have appeared to influence the work reported in this paper.
